# Renal dysfunction in people with hidradenitis suppurativa: a multi-center, propensity-score-matched cohort study

**DOI:** 10.7150/ijms.102434

**Published:** 2025-01-01

**Authors:** Shuo-Yan Gau, Chi-Ya Yang, Yun-Feng Li, Chien-Ying Lee, Yu-Jung Su, Hui-Chin Chang, Meng-Che Wu

**Affiliations:** 1 Department and Graduate Institute of Business Administration, National Taiwan University, Taipei, Taiwan.; 2 Department of Pharmacology, Chung Shan Medical University, Taichung, Taiwan.; 3 Orthopedics Department, Chi-Mei Medical Center, Tainan, Taiwan.; 4 Chang Gung Memorial Hospital, Linkou branch, No. 5, Fuxing St., Guishan Dist., Taoyuan City, 33305, Taiwan.; 5 Bloomberg School of Public Health, Johns Hopkins University, 615 N. Wolfe Street, Baltimore, Maryland, United States.; 6 School of Medicine, Chung Shan Medical University, Taichung, Taiwan.; 7 Department of Pharmacy, Chung Shan Medical University Hospital, Taichung, Taiwan.; 8 Evidence-based Medicine Center, Chung Shan Medical University Hospital, Taichung, Taiwan.; 9 Library, Chung Shan Medical University Hospital, Taichung, Taiwan.; 10 Division of Pediatric Gastroenterology, Children's Medical Center, Taichung Veterans General Hospital, Taichung, Taiwan.; 11 Department of Post-Baccalaureate Medicine, College of Medicine, National Chung Hsing University, Taichung, Taiwan.

**Keywords:** hidradenitis suppurativa, acute kidney injury, chronic kidney disease, cohort, epidemiology, electronic medical records

## Abstract

**Background:** Recent studies suggest a potential link between HS and renal dysfunction. Our objective is to assess the correlation between hidradenitis suppurativa (HS) and renal consequences, specifically focusing on acute kidney injury (AKI), chronic kidney disease (CKD), and end-stage renal disease (ESRD).

**Methods:** This study was performed based on retrospective cohort design. Electronic medical records of participants were retrieved from the US collaborative network in the TriNetX research network. Information from 46,561 individuals with HS was examined alongside an equivalent number of matched controls. Propensity matching was performed for matching confounders. The study spanned from January 1, 2005, to December 31, 2017. Primary outcomes were set as renal dysfunction, including AKI, CKD, and ESRD.

**Results:** Over the 1-year follow-up, people with HS presented a 1.84-fold higher risk of AKI (95% CI, 1.34-2.53) and a 1.37-fold higher risk of CKD (95% CI, 1.02-1.85) than non-HS individuals. Elevated risks persisted over the longer follow-up periods for AKI at 1.51-fold (95% CI, 1.28-1.77) for 3-years-follow-up and 1.47-fold (95% CI, 1.30-1.65) for 5-years-follow-up, respectively. Stratification by sex revealed higher risks in males, and comparison with psoriasis patients indicated increased AKI and CKD risks in HS patients.

**Conclusion:** This study highlights a significant association between HS and renal dysfunction, emphasizing the need for further exploration of shared pathophysiological mechanisms. The findings could offer potential insights into HS-related comorbidities.

## Introduction

Hidradenitis suppurativa (HS) is a long-lasting inflammatory disease that targets areas of the body with apocrine glands, characterized by deep nodular growths, pus-filled abscesses, abnormal fistula tracts, and scarring in the perianal and axillary regions^1^-^3^. Studies have reported a high burden of comorbidities associated with HS, including endocrinological disorders, inflammatory diseases, and cardiovascular outcomes^4^-^7^. Common treatments include surgery, topical therapies (antibiotic or non-antibiotic), systemic antibiotics, and biologics^8^.

Comorbidities such as diabetes and cardiovascular diseases are linked to chronic kidney disease. An inpatient study by Almuhanna *et al.* showed that patients with HS had a higher adjusted odds ratio of chronic kidney disease compared to non-HS patients^9^. Genetic susceptibility, immune dysfunction, and cytokine dysregulation have been suggested as contributors to the relationship between HS and renal diseases^2^; ^10^. HS, as a chronic or relapsing disease involving the Th17/IL-17 axis, promotes fibrosis, loss of organ architecture, and function, which could lead to podocyte damage and renal dysfunction, such as acute kidney injury (AKI) or chronic kidney disease (CKD) 11.

We aim to further investigate these findings using the TriNetX research network. Our study would be the first of its kind to use a database that includes more than 60 healthcare organizations in the United States, encompassing health records from over 75 million patients, to examine the relationship between HS and renal dysfunction.

## Materials and Methods

### Utilized database for analysis

This study was performed based on retrospective cohort design. The dataset for this study was obtained from the TriNetX research network, a globally federated research platform that is prospectively updated. TriNetX offers data derived from de-identified electronic health records contributed by its collaborative healthcare organizations (HCOs). As of now, TriNetX encompasses over 120 HCOs spanning countries across the Americas, Europe, and Asia. For our analysis, we utilized the US collaborative network, a subset of TriNetX extensively applied in the fields of clinical epidemiology and dermatology research 12-16. This subset specifically collects data from HCOs based in the United States and comprises 60 HCOs, encompassing health records from over 75 million patients. This study follows the STROBE guidelines for reporting, and inform consent were waived due to the de-identification process of the TriNetX database.

### Study population

This research was conducted over a period from the start of 2005 to the end of 2017. The HS group consisted of people who had recorded visits and were diagnosed with HS. On the other hand, the control group, labeled as non-HS, was made up of individuals who had undergone health checks and had no prior HS diagnosis. Both groups excluded individuals younger than 18, those with a history of tumors, acute kidney injury (AKI), chronic kidney disease (CKD), end-stage renal disease (ESRD), or kidney failure, and those who passed away before the reference date. To create a control group that could be compared in later analysis, we carried out a 1:1 propensity score matching process.

### Study settings and covariates

The main analysis considered potential confounders such as age, sex, race, body mass index (BMI), comorbidities (including diabetes mellitus, hypertension, systemic lupus erythematosus), medication usage (antibiotics, corticosteroids, immunomodulators), mental and behavioral disorders due to psychoactive substance use, medical utilization status, laboratory data on renal function (creatinine, blood urea nitrogen, eGFR), and socioeconomic status as matching covariates. After matching, we included 46,561 people diagnosed with HS and a same amount of non-HS controls without HS. The main results were identified as kidney-related complications, encompassing AKI, CKD, and ESRD. With the help of the forward-looking update function in TriNetX, all study participants were monitored for at least a 5-year period. The codes used in this research are detailed in **Table S1**.

### Subgroup and sensitivity analyses

Analyses of subgroups were performed by dividing the population into various subgroups based on age and gender. Sensitivity analyses were performed using various matching algorithms and applying wash-out periods to address potential reversed causality. In specific wash-out periods, incident events occurring within that period were not considered as outcome events. Additionally, recognizing the reported positive association between psoriasis and renal dysfunction^17^, we assessed the risk of future renal diseases in HS patients, setting the control group as individuals with psoriasis.

### Statistical analysis

We utilized TriNetX's analytic function to perform all statistical evaluations. We estimated hazard ratios (HR) to compare the risk of new outcome events between HS and their corresponding control groups. For each evaluation, we computed 95% confidence intervals (95% CI) to ascertain the statistical significance of our findings. We employed the Standardized Difference (SD) to display the baseline attributes of covariates pre and post propensity score matching. An SD value below 0.1 signifies a negligible disparity between the groups.

## Results

**Figure 1:**
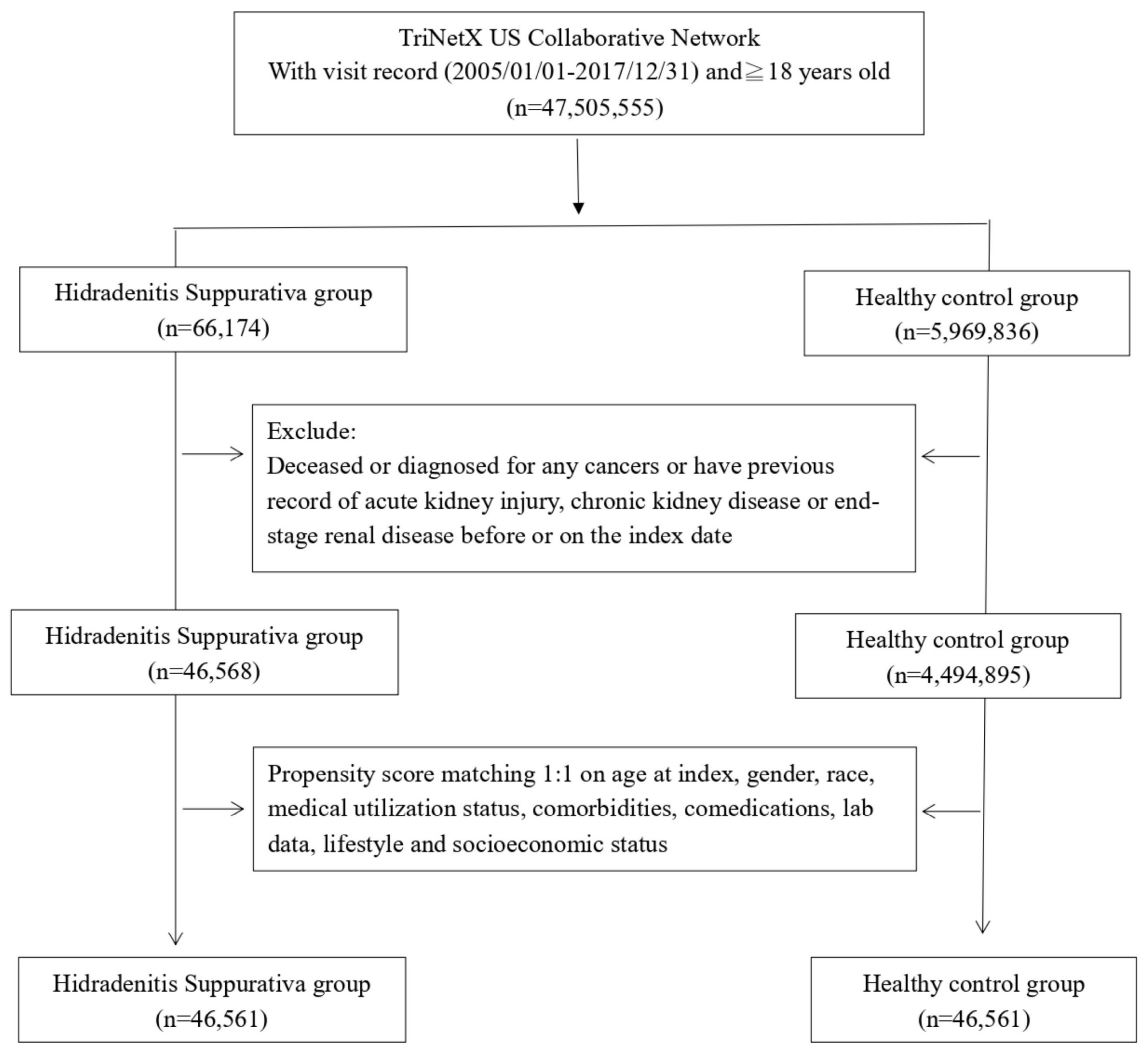
Patient selection process.

Before matching, we included 45,568 HS patients and 4,494,895 non-HS patients for analysis (**Fig. 1**). Initial attributes such as age, gender, ethnicity, lifestyle habits, and concurrent medication usage exhibited notable disparities between the two cohorts. However, post-matching, these variations in initial attributes were no longer statistically significant (**Table 1**).

In the first year of observation, HS patients exhibited a risk of AKI that was 1.84 times greater than their non-HS counterparts (95% CI, 1.34-2.53). The likelihood of CKD was also higher in HS patients, at 1.37 times that of non-HS individuals (95% CI, 1.02-1.85). This elevated risk of AKI persisted over the course of 3 and 5 years, with a 1.51-fold (95% CI, 1.28-1.77) and 1.47-fold (95% CI, 1.30-1.65) increase, respectively. As for the development of CKD, HS patients had a risk that was over 25% higher than non-HS patients. These significant correlations between HS, AKI, and CKD remained consistent in sensitivity models that utilized various matching covariates for propensity score matching and wash-out periods (**Table 2, Table S2-S3**). The risk of ESRD in HS patients was found to be not significant during the follow-up periods of 1 year, 3 years, and 5 years, according to both the primary analysis and the sensitivity models (**Table 2, Table S2-S3**). Relative to those diagnosed with psoriasis, the likelihood of HS patients experiencing AKI and CKD was 1.24 (95% CI, 1.10-1.41) and 1.21 (95% CI, 1.06-1.37), respectively (**Table S4**).

Upon stratified analysis, it was found that male patients with HS had a risk of developing AKI that was 1.63 times higher (95% CI, 1.34-2.00), whereas the risk for female patients was 1.31 times higher (95% CI, 1.13-1.51) over a span of 5 years. In age stratification, although individuals between 18 and 64 years old showed a significant association with AKI risk, those aged greater than 65 years presented a neutral association with AKI risk (HR=1.19; 95% CI, 0.93-1.51) (**Table 3**). The significance of CKD risk in HS patients was observed in different age and sex stratification subgroups. HS patients who were male had a 35% increased probability of future CKD development (HR=1.35; 95% CI, 1.10-1.66), while female HS patients had a risk that was 26% greater than the control group (HR=1.26; 95% CI, 1.08-1.46). Nonetheless, in terms of the risk for ESRD, there was no statistical significance found in stratifications based on age and sex (**Table 4**).

## Discussion

In our research, the HS group demonstrated significantly elevated hazard ratios for both AKI and CKD compared to the non-HS group. The hazard ratios were especially higher during the early years of follow-up, and declined with longer follow-up periods, though all with higher risk. This is supported by research that has found upregulated monocyte release of IL-1 that increased systemic inflammatory load, and vascular dysfunction that could lead to renal dysfunction^18^; ^19^. IL-12 and IL-23 were found to be abundantly expressed by macrophages, with the IL-23/Th17 pathway being crucially involved in the pathogenesis of HS, leading to chronic inflammation of this debilitating disease^20^.

In our investigation, it was found that the risk for AKI and CKD was higher in male HS patients compared to females. The probability of CKD development in the future was 35% higher for male HS patients (HR=1.35; 95% CI, 1.10-1.66), while for female HS patients, the risk was 26% greater than the control group (HR=1.26; 95% CI, 1.08-1.46). However, no significant difference was observed in the risk of ESRD when stratified by age and sex. Although CKD is generally more prevalent in women worldwide, men have a higher incidence of end-stage kidney failure, especially those undergoing kidney replacement therapy^21^. These findings could indicate that the presence of HS may increase the prevalence of CKD in males. Also, it should be noted that males are more likely than females to have severe HS disease and therefore increasing the risk of renal damage in males^22^.

We also found a greater risk for HS patients developing AKI and CKD than psoriasis patients. It has been studied that HS has a higher inflammatory profile than psoriasis that could possible explain higher likelihood of AKI and CKD of HS due to more chronic inflammation^23^. HS and psoriasis can both result in glomerular impairment, which can lead to CKD and glomerulonephritis often seen in autoimmune diseases^24^. At the same time, it has been identified that psoriasis could be a potential comorbidity of HS and was reported to have high prevalence in HS patients 25; 26. Despite differences in skin manifestation of the two inflammatory disorders HS and psoriasis, both can develop renal dysfunction such as CKD. Recently, case reports suggest that treating HS in patients with renal dysfunction may be feasible. A case from Germany described a 57-year-old woman on hemodialysis for end-stage renal disease who was treated with secukinumab, resulting in improvements in pain, inflammation, and disease severity, with no reported adverse effects 11. Another case from Italy involved a 24-year-old male with juvenile nephronophthisis on chronic dialysis. After previous treatments failed, he showed improvement in quality of life, pain reduction, and HS activity following secukinumab treatment​^27^. Similarly, a 53-year-old man from Turkey with HS, amyloidosis, sepsis, and acute renal failure was treated with TNF inhibitors like adalimumab, which helped manage his HS complications despite persistent renal impairment​^28^. These cases indicate that biologic treatments like secukinumab and TNF inhibitors may offer potential therapeutic options for HS patients with renal dysfunction.

Our study, while robust in its population size, is subject to certain limitations. Firstly, as in other database-based observational studies, the causation between the intervention and the outcome events could not be determined^29^; ^30^. In this present research, the discerned real-world correlation between HS and kidney impairment should be interpreted with caution. Secondly, given that clinical indexes such as Hidradenitis Suppurativa Physician's Global Assessment scale (HS-PGA) or International Hidradenitis Suppurativa Severity Score System (IHS4) were not available in the database, we were unable to precisely define and further classify the severity of HS of each individual in the study. Thirdly, there can still be misclassification bias or unaccounted confounders despite matching and sensitivity analyses^31^.

In conclusion, our study suggests significant associations between HS and renal dysfunction, providing valuable insights into the relationship between these conditions. Future studies would be needed to contribute to the understanding of these associations with HS that can impact clinical practice.

## Supplementary Material

Supplementary tables.

## Figures and Tables

**Table 1:** Baseline characteristics of study subjects (before and after propensity score matching)

	Before matching				After matching^a^		
	HS cohort (n=46,568)	Control cohort (n= 4,494,895)	Std. diff.		HS cohort (n=46,561)	Control cohort (n=46,561)	Std. diff.
**Age at index**							
Mean±SD	32.6±13.6	36.1±20.0	**0.21**		32.6±13.7	33.1±14.2	0.04
**Sex**							
Male	11040(23.7)	1899881(42.3)	**0.40**		11040(23.7)	11046(23.7)	0.00
Female	34864(74.9)	2462755(54.8)	**0.43**		34857(74.9)	34908(75.0)	0.00
**Race, n (%)**							
White	20523(44.1)	2649859(59.0)	**0.30**		20523(44.1)	20433(43.9)	0.00
Black or African American	15963(34.3)	662072(14.7)	**0.47**		15956(34.3)	16170(34.7)	0.01
Asian	764(1.6)	155764(3.5)	**0.12**		764(1.6)	1419(3.0)	0.09
American Indian or Alaska Native	205(0.4)	14687(0.3)	0.02		205(0.4)	166(0.4)	0.01
**Socioeconomic status**							
Socioeconomic/psychosocial circumstances problem	928(2.0)	30183(0.7)	**0.12**		924(2.0)	826(1.8)	0.02
**Lifestyle**							
Alcohol dependence, smoking and substance use	5221(11.2)	127342(2.8)	**0.33**		5214(11.2)	5322(11.4)	0.01
**Comorbidities**							
Hypertension	5159(11.1)	366187(8.2)	**0.10**		5156(11.1)	5088(10.9)	0.00
Diabetes mellitus	2847(6.1)	142757(3.2)	**0.14**		2842(6.1)	2781(6.0)	0.01
Hyperlipidemia	2729(5.9)	246390(5.5)	0.02		2728(5.9)	2622(5.6)	0.01
Systemic lupus erythematosus	176(0.4)	5347(0.12)	0.05		175(0.4)	136(0.3)	0.01
**Medications**							
Penicillins and beta-lactam antimicrobials	10284(22.1)	448180(10.0)	**0.33**		10277(22.1)	10210(21.9)	0.00
Aminoglycosides	1487(3.2)	58800(1.31)	**0.13**		1485(3.2)	1457(3.1)	0.00
Corticosteroids	10081(21.6)	501320(11.2)	**0.29**		10074(21.6)	10019(21.5)	0.00
Methotrexate	223(0.5)	7068(0.2)	0.06		221(0.5)	204(0.4)	0.01
Cyclosporine	42(0.1)	3501(0.1)	0.00		42(0.1)	41(0.1)	0.00
**Medical Utilization Status**							
Ambulatory visit	26960(57.9)	2170205(48.3)	**0.19**		26956(57.9)	27461(59.0)	0.02
Inpatient visit	7829(16.8)	498010(11.1)	**0.17**		7828(16.8)	7969(17.1)	0.01
**Laboratory data**							
BMI, n (%)							
≥ 25 (kg/m^2^)	6233(13.4)	291938(6.5)	**0.23**		6226(13.4)	6447(13.8)	0.01
Urea nitrogen, n (%)							
≥ 25 (mg/dL)	418(0.9)	53290(1.2)	0.03		418(0.9)	378(0.8)	0.01
Creatinine, n (%)							
≥ 1.5 (mg/dL)	231(0.5)	16971(0.4)	0.02		231(0.5)	209(0.4)	0.01
Glomerular filtration rate, n (%)							
≥ 90 mL/min/[1.73_m2]	9354(20.1)	388699(8.7)	**0.33**		9347(20.1)	9368(20.1)	0.00

Bold font represents a standardized difference was more than 0.1 HS: Hidradenitis Suppurativa; ^a^ Propensity score matching was performed on age at index, sex, race, body mass index, status of comorbidities (including diabetes mellitus, hypertension, systemic lupus erythematosus), status of comedication use (antibiotics, corticosteroids and immunomodulators), status of smoking, alcoholism and substance use, medical utilization status, lab data regarding renal function status (creatine, blood urea nitrogen, eGFR) and socioeconomic status (problems related to housing and economic circumstances, persons with potential health hazards related to socioeconomic and psychosocial circumstances).

**Table 2:** Risk of renal diseases under different follow-up time^a^

Outcomes	Hazard ratio (95% Confidence interval)^b^		
	1 year	3 years	5 years
Acute kidney injury	**1.84 (1.34,2.53)**	**1.51 (1.28,1.77)**	**1.47 (1.30,1.65)**
Chronic kidney disease	**1.37 (1.02,1.85)**	**1.34 (1.14,1.57)**	**1.27 (1.13,1.44)**
End-stage renal disease	1.68 (0.40,7.02)	1.06 (0.56,2.01)	2.00 (0.71,1.69)

HS: hidradenitis suppurativa ^a^ Data present here were the value of follow up from 90 days after index date to the respective following up years. ^b^ Propensity score matching was performed on age at index, sex, race, body mass index, status of comorbidities (including diabetes mellitus, hypertension, systemic lupus erythematosus), status of comedication use (antibiotics, corticosteroids and immunomodulators), status of smoking, alcoholism and substance use, medical utilization status, lab data regarding renal function status (creatine, blood urea nitrogen, eGFR) and socioeconomic status (problems related to housing and economic circumstances, persons with potential health hazards related to socioeconomic and psychosocial circumstances).

**Table 3:** Stratification analysis of acute kidney injury risk in HS patients

	Cases occurring new-onset acute kidney injury		
Subgroups	HS cohort (No. of event/HS patient amount in each subgroup)	Control cohort (No. of event/non-HS patient amount in each subgroup)	HR (95% CI)^a^
**Gender**			
Male	260/11040	162/11040	**1.63 (1.34,2.00)**
Female	423/34858	319/34858	**1.31 (1.13,1.51)**
**Age at index date**			
18-64 years old	564/42957	397/42957	**1.41 (1.24,1.60)**
≥ 65 years old	142/3608	123/3608	1.19 (0.93,1.51)

^a^ Propensity score matching was performed on age at index, sex, race, body mass index, status of comorbidities (including diabetes mellitus, hypertension, systemic lupus erythematosus), status of comedication use (antibiotics, corticosteroids and immunomodulators), status of smoking, alcoholism and substance use, medical utilization status, lab data regarding renal function status (creatine, blood urea nitrogen, eGFR) and socioeconomic status (problems related to housing and economic circumstances, persons with potential health hazards related to socioeconomic and psychosocial circumstances). ^b^ In order to protect the privacy of participants, the TriNetX system was not able to present the exact number of participant if the number was less than 10. Hence for these stratification groups, we were not able to calculate the hazard ratio.

**Table 4:** Stratification analysis of chronic kidney disease and end-stage renal disease risk in HS patients

	New-onset chronic kidney disease			New-onset end-stage renal disease			
Subgroups	HS cohort (No. of event/ HS patient amount in each subgroup)	Control cohort (No. of event/ non-HS patient amount in each subgroup)	HR (95% CI)^a^	HS cohort (No. of event/ HS patient amount in each subgroup)	Control cohort (No. of event/ non-HS patient amount in each subgroup)	HR (95% CI)^a^	
**Sex**							
Male	208/11040	156/11040	**1.35 (1.10,1.66)**	NA	NA	NA	
Female	379/34858	298/34858	**1.26 (1.08,1.46)**	30/34858	32/34858	0.92 (0.56,1.52)	
**Age at index date**							
18-64 years old	390/42957	293/42957	**1.32 (1.13,1.54)**	34/42957	35/42957	0.96 (0.60,1.54)	
≥ 65 years old	209/3608	172/3608	**1.26 (1.03,1.54)**	NA^b^	NA	NA	

^a^ Propensity score matching was performed on age at index, sex, race, body mass index, status of comorbidities (including diabetes mellitus, hypertension, systemic lupus erythematosus), status of comedication use (antibiotics, corticosteroids and immunomodulators), status of smoking, alcoholism and substance use, medical utilization status, lab data regarding renal function status (creatine, blood urea nitrogen, eGFR) and socioeconomic status (problems related to housing and economic circumstances, persons with potential health hazards related to socioeconomic and psychosocial circumstances). ^b^ To protect the privacy of participants, the TriNetX system does not provide the exact number of participant if the number is < 10. Hence for these stratification groups, we were not able to calculate the hazard ratio.
