# Supplementary material for: Renal dysfunction in people with hidradenitis suppurativa: a multi-center, propensity-score-matched cohort study
Source: Int J Med Sci. 2025 Jan 1;22(3):558–64. doi: 10.7150/ijms.102434 (PMC11783075; doi:10.7150/ijms.102434)
Supplement: Supplementary file 1 — Supplementary tables. [file ijmsv22p0558s1.pdf]

Supplementary Files

Supplementary Tables

Table S1. Utilized adminstrative codes in the current study

**Description**	**ICD-10-CM codes [a]**
Hidradenitis suppurativa	L73.2
Neoplasms	C00-D49
Acute kidney injury	N17
Chronic kidney disease	N18
End-stage renal disease	N18.6
Diabetes mellitus	E08-E13
Hypertension	I10
Hyperlipidemia	E78.5
Systemic lupus erythematosus	M32
Health hazards related to socioeconomic and psychosocial circumstances	Z55-Z65
Encounter for health examinations	Z00
Substance abuse	F10-F19
**Medications**	**RxNorm/VA/ATC code**
Penicillins and beta-lactam antimicrobials	VA: AM114
Aminoglycosides	VA: AM300
Corticosteroids	ATC code: D07
Methotrexate	RxNorm: 6851

Cyclosporine	RxNorm: 3008

[a]ICD-10-CM: International Classification of Diseases, Tenth Revision, Clinical Modification

Table S2. Sensitivity analysis: risk of renal diseases in HS patients based on different covariate matching models, with 5-year follow up

Outcomes	Hazard ratio (95% Confidence interval)			
	Crude	Model 1[a]	Model 2[b]	Model 3[c]
Acute kidney injury	**1.53 (1.42,1.65)**	**2.14 (1.88,2.44)**	**1.72 (1.52,1.94)**	**1.99 (1.75,2.26)**
Chronic kidney disease	0.91 (0.84,0.99)	**1.64 (1.43,1.87)**	**1.34 (1.18,1.52)**	**1.63 (1.43,1.85)**
End-stage renal disease	1.21 (0.90,1.636)	1.21 (0.77,1.90)	0.94 (0.62,1.43)	**1.26 (0.80,1.98)**

[b] Propensity score matching was performed on age at index, sex, race and BMI

[b] Propensity score matching was performed on age at index, sex, race and comorbidities

[c] Propensity score matching was performed on age at index, sex, race and eGFR

[d] Propensity score matching was performed on age at index, sex, race, medical utilization status and socioeconomic status

Table S3. Sensitivity analysis: risk of renal diseases with different wash-out periods [a]

Outcomes	Hazard ratio (95% Confidence interval)[b]		
	12 months	24 months	36 months
Acute kidney injury	**1.41 (1.25,1.59)**	**1.39 (1.22,1.59)**	**1.44 (1.24,1.67)**
Chronic kidney disease	**1.27 (1.12,1.43)**	**1.23 (1.08,1.41)**	**1.26 (1.10,1.46)**
End-stage renal disease	1.15 (0.74,1.79)	1.05 (0.65,1.69)	1.06 (0.64,1.75)

[a] Incident events occurred within each wash-out period were excluded in the corresponding analysis, following up for 5 years after index date

[b] Propensity score matching was performed on age at index, sex, race, body mass index, status of comorbidities (including diabetes mellitus, hypertension, systemic lupus erythematosus), status of comedication use (antibiotics, corticosteroids and immunomodulators), status of smoking, alcoholism and substance use, medical utilization status, lab data regarding renal function status (creatine, blood urea nitrogen, eGFR) and socioeconomic status (problems related to housing and economic circumstances, persons with potential health hazards related to socioeconomic and psychosocial circumstances).

Table S4. Sensitivity analysis: risk of renal diseases comparing with psoriasis patients [a]

Outcomes	Hazard ratio (95% Confidence interval)[b]
Acute kidney injury	**1.24 (1.10,1.41)**
Chronic kidney disease	**1.21 (1.06,1.37)**
End-stage renal disease	1.38 (0.83,2.28)

[a] Data present here were the value of follow up from 90 days after index date to the respective following up years.

[b] Propensity score matching was performed on age at index, sex, race, body mass index, status of comorbidities (including diabetes mellitus, hypertension, systemic lupus erythematosus), status of comedication use (antibiotics, corticosteroids and immunomodulators), status of smoking, alcoholism and substance use, medical utilization status, lab data regarding renal function status (creatine, blood urea nitrogen, eGFR) and socioeconomic status (problems related to housing and economic circumstances, persons with potential health hazards related to socioeconomic and psychosocial circumstances).
